# Trends in Antibiotic Self-Medication for Dental Pathologies among Patients in the Dominican Republic: A Cross-Sectional Study

**DOI:** 10.3390/jcm10143092

**Published:** 2021-07-13

**Authors:** Juan Manuel Aragoneses, Javier Aragoneses, Cinthia Rodríguez, Juan Algar, Ana Suárez

**Affiliations:** 1 Faculty of Dentistry, Universidad Alfonso X El Sabio, 28691 Madrid, Spain; jmaragoneses@gmail.com; 2 Department of Medicine and Medical Specialties, Faculty of Health Sciences, University of Alcalá, 28801 Madrid, Spain; javias511@gmail.com; 3 Department of Dentistry, Universidad Federico Henriquez y Carvajal, Santo Domingo 10106, Dominican Republic; cinthiagarabitos@gmail.com; 4 Department of Clinical Dentistry, School of Biomedical Sciences, Universidad Europea de Madrid, 28005 Madrid, Spain; juan.algar2@universidadeuropea.es; 5 Department of Preclinical Dentistry, School of Biomedical Sciences, Universidad Europea de Madrid, 28670 Madrid, Spain

**Keywords:** antibiotics, bacterial resistance, self-medication, dental pathology, Dominican Republic

## Abstract

The World Health Organisation has warned of the increase in antibiotic resistance, estimating that by 2050 it could become the leading cause of death worldwide. Several studies and literature reviews show a correlation between antibiotic use and bacterial resistance, with unnecessary broad-spectrum antibiotics, such as amoxicillin/clavulanic acid and azithromycin, being one of the main causative factors. An interview-based survey of 2160 participants was conducted to assess the prevalence in the practice of self-medication with antibiotics among dental patients in the Dominican Republic. A series of open-ended questions regarding self-medication and class of antibiotics were put to the patients. Over a third of the study population (39.7%) admitted to the practice of antibiotic self-medication. Most of the respondents (58.4%) who indulged in self-medication were females, and it was prevalent in the older adults between 32–42 years old (36%). There was a negative correlation between age and self-medication practice (*p* < 0.001) observed with point biserial correlation test. Binary logistic regression analysis found an odds ratio of 0.97 (*p* < 0.001). The most consumed antibiotics were amoxicillin/clavulanic acid (52%), amoxicillin (31.1%), and azithromycin (10.1%). It is imperative to educate patients about the harmful effects of self-medication and to stress the need for governments to implement stricter laws on non-prescription drug availability.

## 1. Introduction

Bacterial resistance is one of the major problems affecting health care systems [1,2,3], with an estimated 70,000 lives lost each year worldwide [4]. The World Health Organization (WHO) has addressed the issue of antibiotic resistance, stating that there might not be therapeutic options available to treat some bacterial infections that are multidrug-resistant [5], and if this problem is not addressed, the number of deaths is expected to be around 300 million by 2050, and could become the leading cause of death globally [6]. A recent study in the Dominican Republic [7] showed high levels of antibiotic resistance in hospitalized patients, concluding that there is an urgent need to address this growing problem.

Several studies and reviews of existing literature show the correlation between antibiotic use and bacterial resistance [8,9,10,11,12,13,14]. In the fight against the proliferation of antibiotic resistance, it is necessary to emphasize proper infection prevention and control, as well as awareness of the medical team and patients so as to avoid the overuse and misuse of antibiotics, such as self-medication.

It is estimated that in low-income and developing countries, more than 50% of antibiotics can be obtained without prescription from community pharmacies, as they do not have adequate legislation to regulate the sale and distribution of these medicines [15,16,17]. In these countries, the non-prescription availability of antibiotics has a strong correlation with self-medication practices of the population [18]. In addition, Gravningen et al. [19] also warn about the purchase of non-prescription antibiotics during travel abroad, such as in the Dominican Republic.

Two types of self-medication have been identified in the literature: direct and indirect self-medication. Direct self-medication is where patients can directly order the medication based on its generic name, trade name, physical appearance, as well as the consumption of left-over medication from treatment of a previous disease event without prior diagnosis by a professional. Indirect self-medication includes seeking advice from pharmacists or sharing antibiotics with friends and relatives based on their consultation [20,21].

Antibiotic prescription in dentistry is considered to constitute approximately 7–10% [22,23,24] of global antibiotic prescriptions, and it is estimated that 80% of these prescriptions are inappropriate across dentalcare [24,25]. Reasons for engaging in this practice may include the high cost of treatment, poor socioeconomic status, inadequate access to a dental care environment, insufficient insurance coverage [5,26,27,28], cultural influence on some diseases, and dental phobia/anxiety [29]. Considering that many patients self-medicate based on, among others, previous dental experiences or on the recommendation of acquaintances or relatives [30,31], self-medication in dentistry is of great importance to relieve the global burden of antibiotic resistance. Because of the scarce evidence on the self-medication of antibiotics for dental pathologies in the Dominican Republic, the present research aims to obtain data on these practices in order to establish whether it is necessary to strengthen policies at a local level.

## 2. Materials and Methods

This cross-sectional, interview-based survey was conducted in the Clinic of the Faculty of Dentistry, Federico Henriquez y Carvajal University (UFHEC), the Dominican Republic, from 2018 to 2019. The study was approved by the Institutional Review Board of UFHEC (protocol no. 3/10/2017) and written informed consent was obtained from all of the study participants. This survey was conducted with 2160 participants who were chosen based on systematic random sampling of outpatients attending the University Clinic. The sample size was calculated as being 2160, based on a study conducted by Haddadin et al. [32] in 2019, with a power of 95% and an alpha error of 5%.

### 2.1. Eligibility Criteria

The inclusion criteria were as follows: (i) Healthy individuals aged 20 years or older without limit, (ii) patients who had not been previously been treated with antibiotics for this pathology in another dental clinic, (iii) patients who were visiting this dental clinic for the first time with this pathology, and (iv) patients who were not taking antibiotics for other ailments or as prophylaxis. Participants who did not give informed consent, those who were physically or mentally unable to provide objective responses, and children were excluded from this survey.

### 2.2. Survey Instrument

The survey was performed by final year dental undergraduate students (*n* = 50). All were trained and calibrated by a single tutor. The inter-examiner reliability was estimated using the kappa statistics. The survey instrument collected demographic details of the patients such as age and gender, a closed-ended question that elicited information on the practice of antibiotic self-medication, and the type of antibiotic drug used by the patients. The instrument was developed in English; translated and administered in Spanish, the native language of the country; and consistency with back-translation was ensured.

### 2.3. Statistical Analysis

The data were analyzed using IBM SPSS software version 20.0 (IBM.Corp., Armonk NY, USA). The obtained results were subjected to normality tests, such as Kolomogrov−Smirnov and Shapiro−Wilks tests, and the resultant data showed that they followed a parametric distribution. A point biserial correlation analysis was performed to assess the correlation between age, gender, and self-medication practice, with age as a continuous variable and the self-medication practice as a dichotomous variable. A Phi correlation test was used to analyze the correlation between the dichotomous variables, gender and self-medication practice. Binary logistic regression analysis was performed on variables associated with self-medication practice. A *p*-value < 0.05 was considered to be statistically significant and *p*-value < 0.01 was considered to be statistically highly significant.

## 3. Results

This cross-sectional study was performed as an interview-based survey on 2160 enrolled participants. Based on the inclusion criteria, there was a 100% response rate with all the study participants being involved in the survey and clinical examination.

The demographic characteristics of the participants are detailed in Table 1.

Regarding the study of self-medication with antibiotics, 858 patients (39.7%) stated that they practiced self-medication. The demographic characteristics of this group are detailed in Table 2.

A point biserial correlation between variables age, sex, and self-medication practice observed a statistically significant negative correlation between age and the practice (*p*-value < 0.001). The negative correlation indicated that an increase in age resulted in the reduction of self-medication practice among the study population. In addition, the phi correlation test observed a negligible correlation between gender and self-medication practice (Table 3).

When the study on age and the practice of self-medication was carried out by means of binary logistic regression analysis, it was observed that the odds of antibiotic self-medication practice reduced 0.97 times (odd’s ratio [OR] 0.97, *p*-value < 0.001) with the concomitant increase in age of the studied population.

Among the study population who were evaluated for self-reported antibiotic usage for dental pathologies, the most commonly used antibiotic drug for self-medication was Amoxicillin/Clavulanic acid, followed by Amoxicillin, Azithromycin, Clindamycin, and the least (1%) being antibiotics that could not be identified by the study participants (Table 4).

## 4. Discussion

In our survey of 2160 patients, 40% reported self-medication for their dental ailments. These results are consistent with those obtained by Grigoryan et al. [18], who studied self-medication practices in Europe and found that the highest prevalence rates were observed in European countries with a similar culture to the Dominican Republic, such as Spain (25.1–37.9%), Malta (38–46.5%), and Italy (44.4–58.0%). Our data also agree with countries with different cultures, such as Nigeria and Beirut (41.5–42%) [33,34,35], but are lower than in countries across the Indian subcontinent, where reported figures range from 57.3% in Pakistan to 100% in parts of India [30,36,37,38], perhaps because, in this geographical region, almost all pharmacies sell antibiotics without prescription. In contrast, a frequency of self-medication of 23.5% has been reported in Nigeria [12] and 18.7% in Brazil [39], although this does not indicate whether this medication had been previously prescribed by a dentist.

Numerous studies in dentistry [26,33,34,38,40,41] as well as the present research, establish a clear predilection for self-medication among the female gender; however, in our study, there was no statistically significant correlation between variables. According to some authors, the basis for the female predilection for self-medication could be due to a lower pain threshold or fear of dental treatment, as well as the perception that the dental problem is not important [26,33,38,42]. In some countries, because of cultural reasons, women are not allowed to go out unaccompanied to visit a health professional, which, in turn, could imply an increase in self-medication practices in order to treat medical complaints [33]. In contrast, Komal Raj et al. [37] found that self-medication in India was higher among men, especially in middle-aged people, and other studies found no gender predilection [12,43].

When studying a possible relationship between self-medication with age from this survey, it was found to be an important factor in the self-use of antibiotics, because the majority of individuals self-medicating with antibiotics were found to be in the 32–42 age group, compared with younger adults and the older population (>65 years).

These data are consistent with other studies on patients over 18 years, and with dental pathology such as those carried out by Paula et al. [39] in Brazil, Idowu et al. [33] in Nigeria, and Bhattarai et al. [36] in Nepal. This could be because patients in this age group are working age population, and it is possible that a lack of time to seek dental care led them to self-medicate [33,36,37].

However, a study conducted in Saudi Arabia by Dar-Odeh et al. [44] found a wider age range, observing a higher prevalence of self-medication patterns from the age of 25 years to 64 years old. These results are in contrast with a study conducted in Europe that showed that self-medication with antibiotics was higher and more prevalent among the younger population [45]. Older individuals are less likely to use antibiotics without prescription, which may be due to the accumulation of knowledge and experience associated with raising families and ageing.

In the Dominican Republic, there are no established guidelines for the prescription of antibiotics in dentistry that allow for the best choice of antibiotic to be defined. However, if the WHO AWaRe classification is followed [46], antibiotics can be distributed among the following groups: those that minimize the potential for resistance, and therefore are the first choice (Access group); those that are prone to create resistance, and therefore their use should be limited (Watch group); and those that should be reserved as a last resort for cases of multidrug-resistant infections (Reserve group). In the present study, when establishing patterns in relation to the antibiotics most commonly used by self-medicating patients, it was found that the antibiotics most used for self-medication belonged to the Access group (amoxicillin/clavulanic acid and amoxicillin (Access group)), and those least used for this practice belonged to the Whatch (azithromycin) and Access (clindamycin) groups. These results are consistent with the trend observed in the United States, where the most prescribed medications are azithromycin, amoxicillin, amoxicillin/clavulanic acid, cephalexin (Access group), and ciprofloxacin (Watch group) [47]. Amoxicillin is the drug of choice for the treatment of many oral infections [13,48,49,50], and as patients tend to acquire previously prescribed antibiotics, it could explain the higher frequency of penicillin use in this study. These data are also consistent with a study conducted in Saudi Arabia on self-medication with antibiotics, with amoxicillin/clavulanic acid being the most commonly used drug, followed by amoxicillin and metronidazole (Access group) [44], as well as with a study conducted in Brazilian patients, where amoxicillin was also the most frequent antimicrobial [39]. All of these results reaffirm the need to adapt territorial policies to avoid the free availability of antibiotics, especially those that tend to generate more resistance.

Some aspects must be considered regarding data interpretation and extrapolation in this study. First, the survey is a cross-sectional design and does not imply any causal inferences. Although there was a large sample size (*n* = 2160), the generalizability of the study results should be considered with caution, as it was conducted only in one dental care centre and may not be representative of others in the general population of the Dominican Republic. Further research is needed to identify the main factors that favor self-prescribing among patients in need of dental treatment, including weak regulation around easy access and poor regulation for their control and distribution. All of this will help to design solutions to address the misuse of antibiotics, which should include raising public awareness of the problem of antibiotic resistance, as well as regulating access to antibiotics, all to contribute to the sensible use of these drugs.

## 5. Conclusions

Antibiotic misuse through self-medication for dental problems is a problem in the Dominican Republic. Government agencies should ensure that its national action plan to address antibiotic resistance includes addressing the problem of self-medication for dental ailments.

## Figures and Tables

**Table 1:** Demographic characteristics of the study population.

Demographic Details for the Total Population		
Age (Mean ± SD)	21–31 years	449 (20.8%)
	32–42 years	623 (28.8%)
	43–53 years	694 (32.1%)
	54–64 years	366 (16.9%)
	>65 years	28 (1.3%)
Gender (*n* (%))	Male	1262 (58.4%)
	Female	898 (41.6%)

**Table 2:** Frequency and distribution of self-medication practice among the study population according to age and gender.

Variables (*n* = 858)		Frequency (*n*)	Distribution (%)
Age	21–31 years	212	24.7%
	32–42 years	308	35.9%
	43–53 years	260	30.3%
	54–64 years	68	7.9%
	>65 years	10	1.2%
Gender	Female	508	58.4%
	Male	350	41.6%

**Table 3:** Correlation analysis between independent variables age, gender, and practice of self-medication.

Independent Variables		Self-Medication Practice
Age ^1^	Point biserial correlation	−0.143
	*p*-value ^3^	0.001 **
Gender ^2^	Phi correlation	−0.13
	*p*-value ^3^	0.550

^1^ Point biserial correlation test; ^2^ phi correlation test; ^3^ two tailed test; ** *p*-value < 0.001, highly statistically significant.

**Table 4:** Frequency and distribution of the types of antibiotics commonly used in self-medication practice.

Commonly Used Antibiotics	Frequency (*n*)	Distribution (%)
Amoxicillin	267	31.1%
Azithromycin	87	10.1%
Amoxicillin and Clavulanic acid	446	52%
Clindamycin	49	5.7%
Others	9	1%
Total	858	100%
